# Dynamic radiological features predict pathological response after neoadjuvant immunochemotherapy in esophageal squamous cell carcinoma

**DOI:** 10.1186/s12967-024-05291-8

**Published:** 2024-05-18

**Authors:** Yuli Ruan, Yue Ma, Ming Ma, Chao Liu, Dan Su, Xin Guan, Rui Yang, Hong Wang, Tianqin Li, Yang Zhou, Jianqun Ma, Yanqiao Zhang

**Affiliations:** 1 Department of Gastrointestinal Medical Oncology, Harbin Medical University Cancer Hospital, 150 Haping Road, Harbin, Heilongjiang 150001 People’s Republic of China; 2 Department of Radiology, Harbin Medical University Cancer Hospital, 150 Haping Road, Harbin, Heilongjiang 150001 People’s Republic of China; 3 Department of Thoracic Surgery, Harbin Medical University Cancer Hospital, 150 Haping Road, Harbin, Heilongjiang 150001 People’s Republic of China; 4 Translational Medicine Research and Cooperation Center of Northern China, Heilongjiang Academy of Medical Sciences, Harbin, China; 5 Key Laboratory of Tumor Immunology in Heilongjiang, Harbin, China; 6 Clinical Research Center for Colorectal Cancer in Heilongjiang, Harbin, China

**Keywords:** Esophageal cancer, Computed tomography, Neoadjuvant PD-1 blockade, Pathological complete response

## Abstract

**Background:**

Neoadjuvant immunochemotherapy (NICT) plus esophagectomy has emerged as a promising treatment option for locally advanced esophageal squamous cell carcinoma (LA-ESCC). Pathologic complete response (pCR) is a key indicator associated with great efficacy and overall survival (OS). However, there are insufficient indicators for the reliable assessment of pCR.

**Methods:**

192 patients with LA-ESCC treated with NICT from December 2019 to October 2023 were recruited. According to pCR status, patients were categorized into pCR group (22.92%) and non-pCR group (77.08%). Radiological features of pretreatment and preoperative CT images were extracted. Logistic and COX regressions were trained to predict pathological response and prognosis, respectively.

**Results:**

Four of the selected radiological features were combined to construct an ESCC preoperative imaging score (ECPI-Score). Logistic models revealed independent associations of ECPI-Score and vascular sign with pCR, with AUC of 0.918 in the training set and 0.862 in the validation set, respectively. After grouping by ECPI-Score, a higher proportion of pCR was observed among the high-ECPI group and negative vascular sign. Kaplan Meier analysis demonstrated that recurrence-free survival (RFS) with negative vascular sign was significantly better than those with positive (*P* = *0.038*), but not for OS (*P* = *0.310*).

**Conclusions:**

This study demonstrates dynamic radiological features are independent predictors of pCR for LA-ESCC treated with NICT. It will guide clinicians to make accurate treatment plans.

**Supplementary Information:**

The online version contains supplementary material available at 10.1186/s12967-024-05291-8.

## Introduction

Neoadjuvant immunochemotherapy (NICT), which combines anti-PD-1 therapy with chemotherapy, has demonstrated promise in the management of locally advanced esophageal squamous cell carcinoma (LA-ESCC) [1–7]. Clinical trials, such as NICE [8], ESCORT-NEO [5], and KEEP-G03 [9], have demonstrated favorable antitumor effects and the safety of immunotherapies, with reported rates of pathologic complete response (pCR) ranging from 26.7 to 39.2%. Notably, a study conducted at our center by Ma et al. [10] found that 27.4% of patients achieved pCR, while 45.2% attained major pathologic response (MPR). These findings highlight the potential of NICT in reducing tumor burden, eliminating micrometastatic lesions, and enhancing treatment sensitivity, ultimately leading to a promising prognosis.

The pCR, characterized by the complete absence of tumor in surgically excised tissue and lymph nodes following meticulous microscopic examination [11], is a dependable measure of the effectiveness of neoadjuvant therapy [12–14]. Although conventional biomarkers such as PD-L1 CPS and tumor mutation burden (TMB) are crucial indicators of immunotherapy efficacy in ESCC, they do not consistently correlate with pCR [2, 15]. Presently, there is a lack of definitive biomarkers that can reliably forecast the pathological response in locally resectable patients receiving NICT. Therefore, extensive research is urgently needed to find a corresponding marker and address this crucial question.

Computed tomography (CT) scanning is a commonly employed modality for the diagnosis and assessment of solid tumors [16]. The arterial phase images contain valuable information on tumor hemodynamics, which is closely linked to tumor activity and may offer insights into residual tumor survival based on contrast uptake [17–19]. Previous studies have indicated that radiomic features may be associated with the biological characteristics of the tumors [20–25]. For example, 18F-FDG PET/CT parameters exhibited excellent predictive capabilities for tumor activity [26]. The NICE trial revealed a moderate positive correlation (R = 0.600) between the reduction in the longest lesion diameter, as measured by CT, and the rate of pathological regression [8]. These findings suggest that CT imaging features warrant further investigation in this study. Therefore, we aimed to utilize dynamic CT imaging features to predict the likelihood of pCR attainment in patients with LA-ESCC undergoing NICT.

## Methods

### Patients

**Fig. 1:**
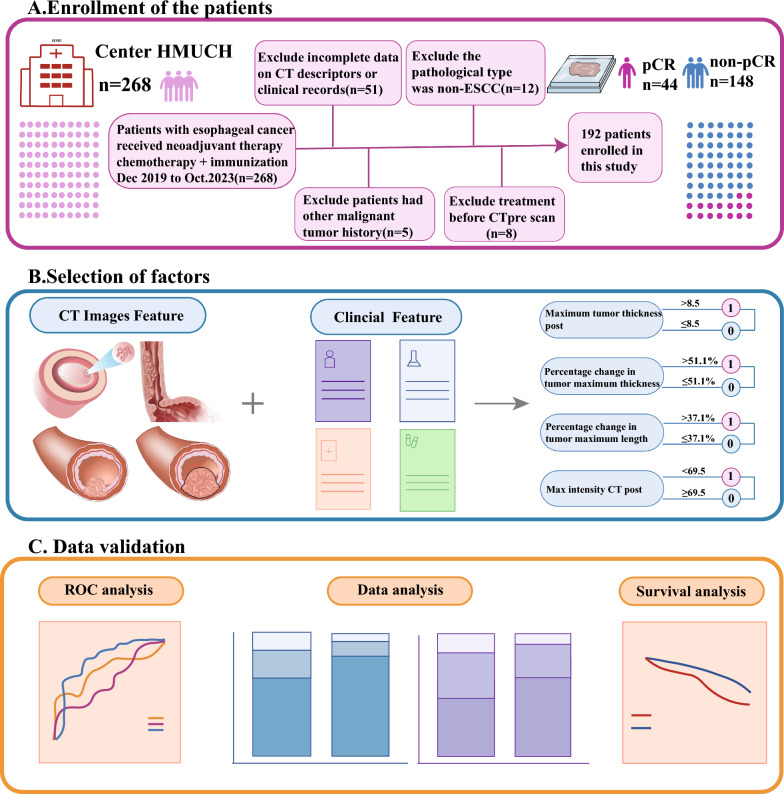
The flow diagram of selection esophageal squamous cell carcinoma patients. **A** Enrollment of patients; **B** selection of factors; **C** data validation. *CT* computed tomography, *ESCC* esophageal squamous cell carcinoma

From December 2019 to October 2023, 268 patients who underwent surgery following NICT for esophageal cancer at Harbin Medical University Cancer Hospital (HMUCH) were included in this study (Fig. 1). The inclusion criteria included individuals aged ≥ 18 years, diagnosed with esophageal cancer staged as cT1b-cT2N+M0 or cT3-cT4a anyNM0, who underwent NICT every three weeks, had contrast-enhanced CT scans before NICT and surgery, and underwent radical esophageal cancer surgery with available pathological findings. Exclusion criteria included incomplete patient imaging or clinical information, non-squamous cell carcinoma revealed in postoperative pathology, presence of other malignancies, and prior therapy before the initial enhanced CT scan. Regular patient follow-ups were conducted, and survival status was recorded until the last follow-up date of November 15, 2023. Recurrence-free survival time (RFS) was defined as the period between surgery and the first recurrence, whereas overall survival (OS) referred to the interval from complete remission post-surgery to death from any cause. In total, 192 patients were retrospectively analyzed, and imaging, pathological, and clinical data processed anonymously to uphold patient confidentiality. Approval for this project was obtained from the ethics committee of HMUCH.

### CT imaging protocol

All dual-phase enhanced CT examinations were conducted using a 64-slice multislice CT scanner (128-slice, Siemens Medical System, Erlangen, Germany), with the following scanning parameters: scanning layer thickness and several reconstructed layers set at 1.25 mm each; 120 kV, 250–300mAs; a 512 × 512 matrix; and image reconstruction at 1 mm. Enhanced image data were obtained by intravenously injecting a non-ionic iodinated contrast agent (iodine concentration: 350 mg/ml) at a rate of 4 ml/s through a contrast syringe. Arterial, portal, and equilibrium phases were obtained at approximately 30–33, 67–70, and 177–180 s, respectively.

### Evaluation of CT features

**Fig. 2:**
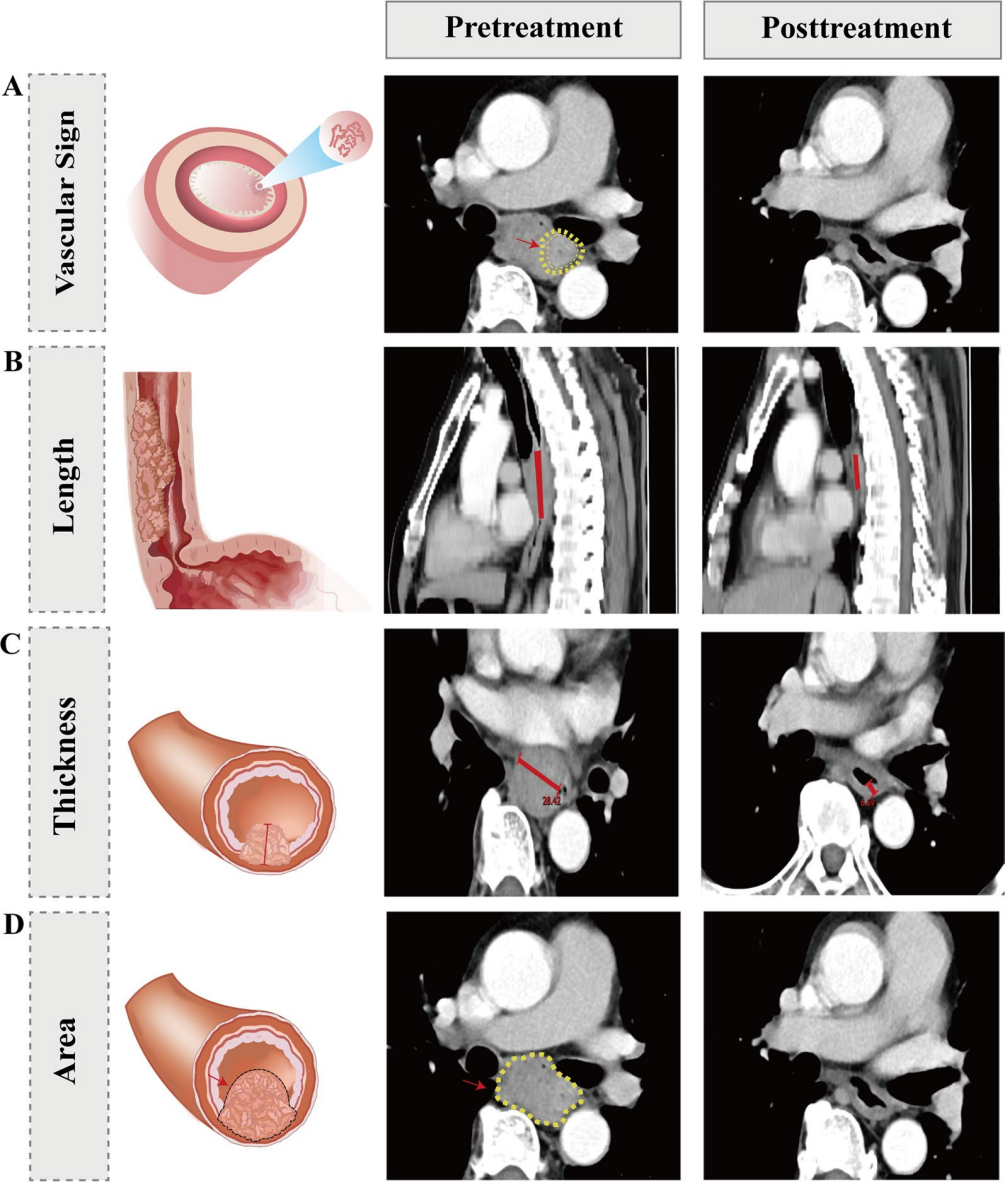
Radiological features before and after treatment for esophageal cancer. **A** Vascular sign; **B** tumor length; **C** tumor thickness; **D** tumor area

Two imaging observers, with 13 and 8 years of experience respectively, conducted a blinded assessment of pretreatment and preoperative CT images using the HMUCH imaging system. The following parameters were documented: maximum thickness and area of the esophageal tumor before treatment and surgery, tumor location, the maximum length of the esophageal tumor using multiple planar reconstructions (MPR), ΔT (difference between pretreatment and preoperative arterial phase tumor attenuation values), ΔTN (difference between arterial phase tumor attenuation values and normal esophageal wall attenuation value), TNR (tumor-to-normal esophageal wall attenuation ratio in the arterial phase), maximum CT value, and vascular sign. The specific meanings of these indicators are detailed in sTable 1, and Fig. 2 provides a schematic diagram illustrating these indicators. CT attenuation values of the tumor were measured using circular regions of interest (ROI) placed on axial sections with the largest tumor diameter, while avoiding ulceration, necrosis, and vascular structures. Background normal esophageal CT attenuation was obtained from ROIs covering the background normal esophageal wall, excluding intraluminal gas, fat, and blood vessels, which are defined as esophagus beyond 5 cm from the tumor.

### Clinical-pathological analysis

Comprehensive clinical-pathological data and thorough follow-up information were collected, including age, gender, BMI, smoking and drinking habits, NICT cycles, clinical T and N stages, tumor location, interval time between preoperative therapy and surgery, pathological T and N stages, lymph node count, and presence of vascular and nerve invasion. The clinical T and N stages of ESCC were determined using endoscopic ultrasound (EUS), and the pathological T and N stages were classified according to the American Joint Committee on Cancer (AJCC) staging system [27]. Patients were divided into pCR and non-pCR groups based on treatment response. The Mandard criteria graded tumor regression (TRG) as follows: TRG 1 (complete regression), TRG 2 (rare residual cancer cells), TRG 3 (fibrosis outgrowing residual cancer cells), TRG 4 (cancer cells outgrowing fibrosis), and TRG 5 (absence of regression) [28, 29].

### Statistical analysis

To compare differences in categorical and continuous variables, the χ^2^ test or Fisher exact tests and T-test or Mann–Whitney test were used. The specificity and sensitivity of various thresholds of continuous variables on survival were assessed using receiver operating characteristic (ROC) curves, with the Youden index employed to determine optimal cutoff values. For variables displaying statistically significant differences, odds ratios (ORs) with 95% confidence intervals (CI) and corresponding P-values were calculated. We performed single and multifactorial logistic regression analyses of classical predictors to identify factors independently associated with pCR. Kaplan–Meier (KM) survival analysis was applied to analyze the OS and RFS. Statistical significance was defined as P < 0.05. Analyses were performed using R (R statistics), version 4.3.2.

## Results

### Patient characteristics

From December 2019 to October 2023, a total of 268 patients underwent surgery after NICT at HMUCH, 76 patients were excluded due to inclusion and exclusion criteria, and last 192 patients were included in this study. Pathological results revealed that 22.92% (44/192) patients achieved pCR, while 77.08% (148/192) did not. Baseline characteristics are summarized in Table 1. There were no significant differences between the pCR and non-pCR groups regarding age, gender, BMI, smoking history, number of pre-surgical treatment cycles, clinical TN stage determined by gastroscopy, interval time between surgery and treatment, and tumor location (*P* > *0.05* for all).

**Table 1:** The clinicopathological features of the enrolled patients

	non-pCR (n = 148)	pCR (n = 44)	*P*.value
Clinicial features			
Age	60.0 (56.0–64.0)	62.0(58.0–66.0)	0.084
Sex			0.544
Female	2 (1.35%)	1 (2.27%)	
Male	146 (98.60%)	43 (97.70%)	
BMI	22.90 (20.50–24.70)	22.60 (21.00–24.80)	0.854
Smoke			0.104
No	68 (45.90%)	27 (61.40%)	
Yes	80 (54.10%)	17 (38.60%)	
Drink			0.021
No	66 (44.60%)	29 (65.90%)	
Yes	82 (55.4%)	15 (34.10%)	
Circle			0.215
1–2	93 (62.80%)	32 (72.70%)	
3–4	46 (31.10%)	12 (27.30%)	
5–6	9 (6.08%)	0	
cT stages			0.156
1	6(4.05%)	0	
2	34 (23.00%)	7 (15.90%)	
3	70 (47.30%)	30 (68.20%)	
4	1 (0.68%)	0	
Unknown	37 (25.00%)	7 (15.90%)	
cN stages			0.085
0	21 (14.20%)	6 (13.60%)	
1	50 (33.80%)	25(56.80%)	
2	38 (25.70%)	6 (13.60%)	
3	2 (1.35%)	0	
Unknown	37(25.00%)	7 (15.90%)	
Interval time	1.33 (1.17–1.54)	1.37 (1.15–1.58)	0.814
Tumor location			0.270
Cardia	9 (6.08%)	1 (2.27%)	
Lower thoracic	84 (56.80%)	30 (68.20%)	
Middle thoracic	46 (31.10%)	13.00 (29.50%)	
Upper thoracic	9(6.08%)	0	
Lymph node size	5.50 (0–10)	10.0 (0–13.00)	0.086
Adjuvant therapy			0.014
No	89 (60.10%)	36(81.80%)	
Yes	59 (39.90%)	8(18.20%)	
Pathological features			
TRG			< 0.001
0	2 (1.35%)	42 (95.50%)	
1	5 (3.38%)	0	
2	13 (8.78%)	0	
3	4(2.70%)	0	
4	12 (8.11%)	0	
5	3 (2.03%)	0	
Unknown	109 (73.60%)	2 (4.55%)	
ypT stages			< 0.001
0	10(6.67%)	43(97.7%)	
1	52(35.1%)	1(2.27%)	
2	20(13.5%)	0	
3	66(44.6%)	0	
ypN stages			< 0.001
0	49(33.1%)	43(97.7%)	
1	65(43.9%)	1(2.27%)	
2	25(16.9%)	0	
3	9(6.08%)	0	
Total lymph nodes removed	35.00 (26.00–49.00)	33.00 (24.00–53.20)	0.813
Number of metastatic lymph	1.00 (0–2.00)	0	< 0.001
Vascular invasion			0.575
No	144 (97.30%)	44 (100%)	
Yes	4(2.70%)	0	
Nerve invasion			1
No	145(98.00%)	44 (100%)	
Yes	3(2.00%)	0	

*Cycle* the number of chemotherapy cycles before surgery, *cT stages* clinical tumor staging, *cN stages* clinical lymph node staging, *Interval time* the interval between the end of the last chemotherapy and surgery, *TRG* tumor regression grade, *ypT satges* tumor staging after neoadjuvant therapy, *ypN satges* lymph node staging after neoadjuvant therapy

### Novel preoperative imaging score for pathological response prediction

**Fig. 3:**
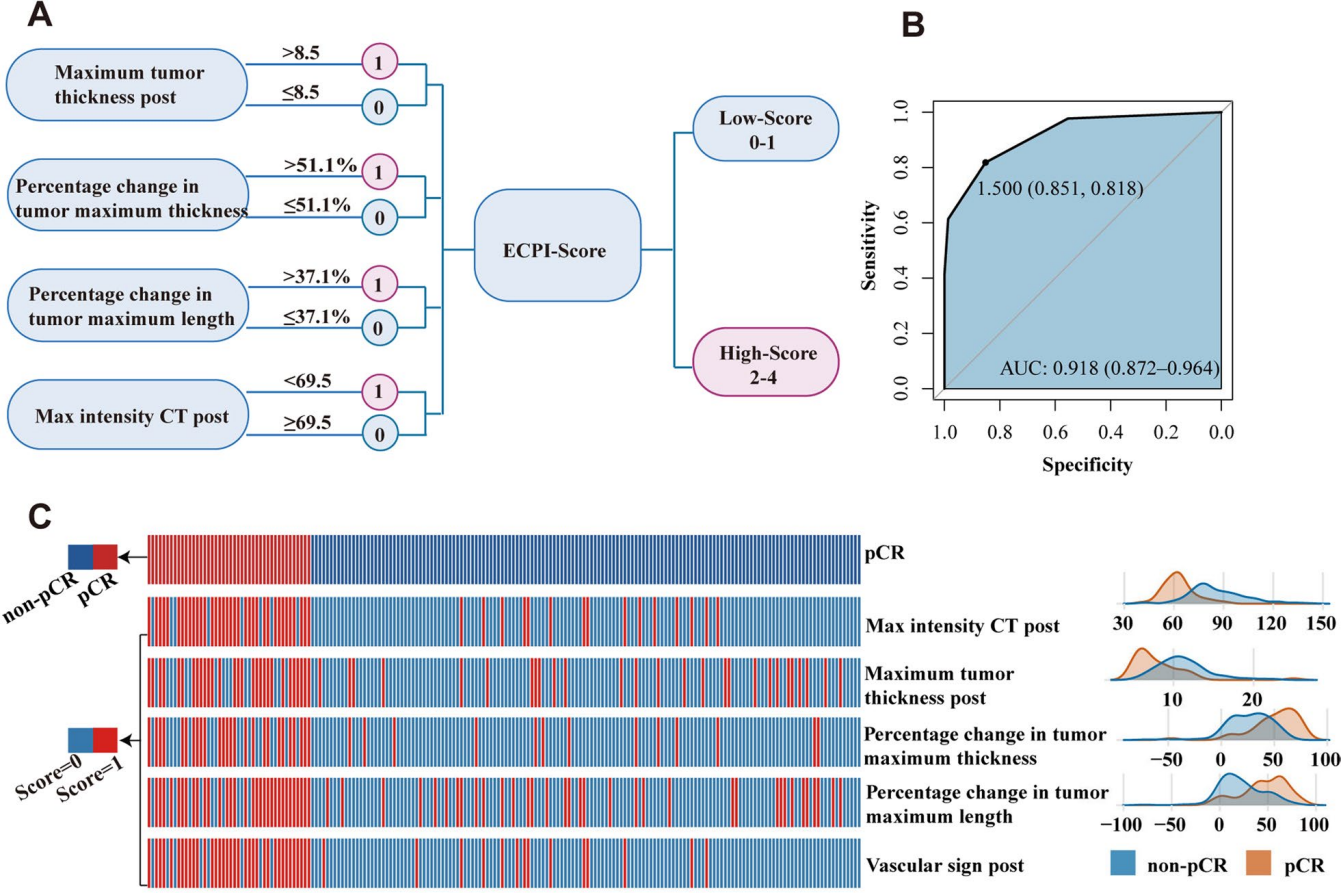
Construction and validation of a scoring system for LA-ESCC. **A** Radiographic scoring system; **B** ROC curve for scoring system; **C** correlation heatmap of individual metrics and pCR in LA-ESCC

**Fig. 4:**
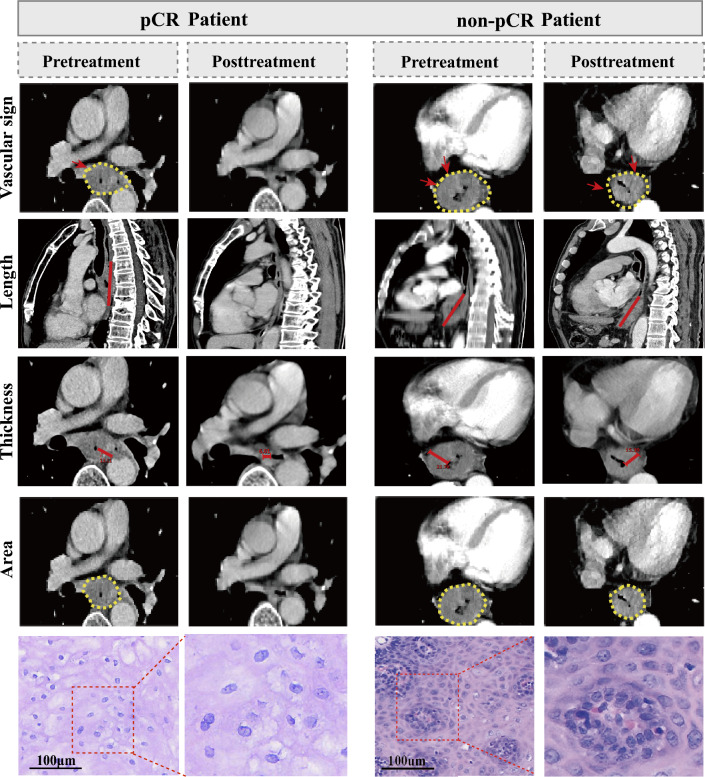
The representative cases of vascular sign and radiological features and pathological tissue in two patients

Statistically significant differences (*P* < *0.001*) were observed in radiological features between the pCR and non-pCR groups, including tumor thickness, length, area, and CT values in Table 2. Optimal classification thresholds for these metrics were displayed in sTable2. Utilizing four metrics with an AUC > 0.75, we developed a novel scoring system, termed ESCC preoperative imaging score (ECPI-Score), including post-treatment maximal CT value, the percentage change in tumor thickness, post-treatment maximal tumor thickness, and the percentage change in maximal tumor length (AUC = 0.900, 0.810, 0.808 and 0.765, respectively) (Fig. 3A). This scoring system demonstrated superior predictive value for preoperative pCR assessment (AUC = 0.918) compared to individual predictors, as depicted in Fig. 3B. The scores were further categorized into a high-ECPI group (≥ 2 scores) (30.21%) and a low-ECPI group (< 2 scores) (69.79%) according to the Youden index. The heatmap of correlation analysis between radiological features and pCR proportions is depicted in Fig. 3C, indicating patients achieving pCR showed higher scores of radiological features than those without pCR. Additionally, two typical patients represent the achievement of pCR versus non-pCR after undergoing NICT, illustrating changes in vascular sign, tumor length, tumor thickness, and tumor area before and after NICT treatment (Fig. 4).

**Table 2:** The imaging parameters of all enrolled ESCC patients

	Non-pCR (n = 148)	pCR (n = 44)	*P.*value
Maximum tumor thickness pre	15.50 (12.00–19.20)	16.50(12.00–22.00)	0.197
Maximum tumor thickness post	11.00 (9.00–13.00)	7.00 (6.00–9.00)	< 0.001
Percentage change in tumor maximum thickness	0.29 (0.12–0.42)	0.56 (0.41–0.67)	< 0.001
Maximum tumor length pre	56.00 (45.80–70.00)	54.00 (44.80–74.50)	0.764
Maximum tumor length post	42.50 (30.00–56.00)	27.00 (22.00–36.00)	< 0.001
Percentage change in tumor maximum length	0.19 (0.06–0.36)	0.47 (0.36–0.62)	< 0.001
Maximum tumor area pre	548.00 (377.00–739.00)	673.00 (423.00–826.00)	0.346
Maximum tumor area post	252.00 (183.00–372.00)	162 (114–246)	< 0.001
Percentage change in tumor maximum area	0.52 (0.34–0.66)	0.72 (0.57–0.80)	< 0.001
CT values pre	58.20 (50.40–64.20)	58.80 (51.90–64.90)	0.586
CT values post	51.80 (44.90–59.00)	43.00 (38.50–50.50)	< 0.001
ΔT	5.50 (− 3.00–13.60)	15.00 (6.50–21.60)	< 0.001
ΔTN post	28.00 (20.40–36.10)	22.20 (15.60–29.90)	0.002
TNR post	2.24 (1.80–2.89)	1.94 (1.64–2.66)	0.054
Max intensity CT pre	88.00 (80.00–99.00)	90.00 (83.50–103.00)	0.188
Max intensity CT post	82.00 (75.00–95.20)	62.50 (56.80–66.00)	< 0.001
ECPI-Score	0 (0–1.00)	3.00 (2.00–4.00)	< 0.001
Vascular sign post			< 0.001
No	14 (9.46%)	36 (81.80%)	
Yes	134(90.50%)	8(18.20%)	

*ESCC* esophageal squamous cell carcinoma, *pCR* pathological complete response, *ΔT* the difference in tumor attenuation values during the arterial phase before treatment and surgery, *ΔTN post* the difference of CT value between tumor and background normal esophageal wall in arterial phase after treatment, *TNR post* CT value ratio of tumor to background normal esophageal wall on arterial phase images after treatment

### Independent factors and clinical correlates of pathological response

**Fig. 5:**
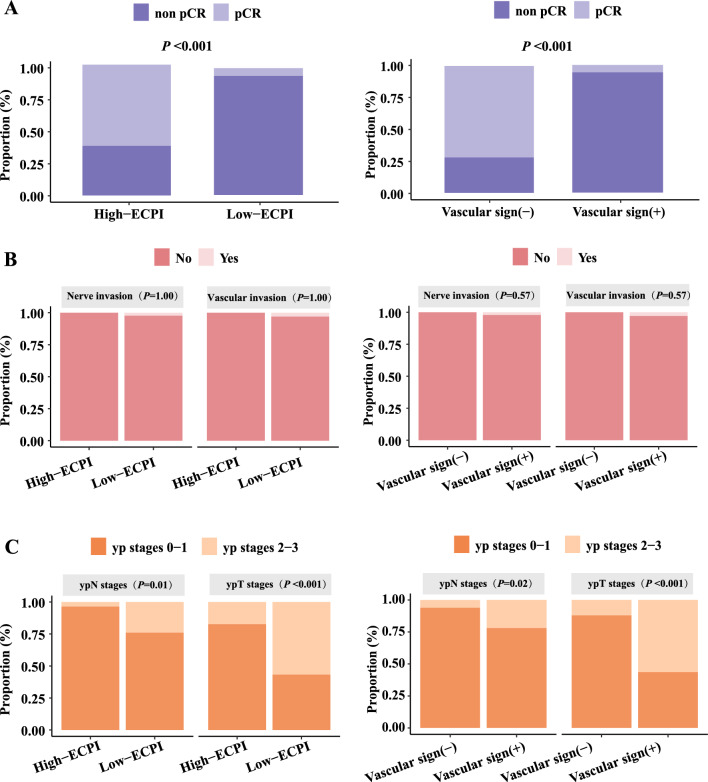
The proportion of each indicator in radiological features **A** pCR vs. non-pCR; **B** nerve invasion vs. vascular invasion; **C** ypT stages vs. ypN stages

We performed logistic regression analysis involving ECPI-Score and vascular sign, alongside other classical predictors to evaluate the correlation with pCR. Both univariate and multivariate analysis revealed that ECPI-Score (OR = 4.28, 95% CI 0.25–74.09, *P* < *0.001*) and vascular sign (OR = 0.12, 95% CI 0.01–2.01, *P* < *0.001*) were independent factors associated with pCR in LA-ESCC patients. The proportion of patients achieving pCR in the high-ECPI group was 62.07%, significantly higher than the 5.97% rates observed among patients in the low-ECPI group (*P* < *0.001*). Similarly, among patients with negative vascular sign, significantly more patients achieved pCR (72.0%) compared to those without (28.00%) (*P* < *0.001*) (Fig. 5A). In indicators related to postoperative pathology, there was no statistically significant difference observed between individuals negative for vascular invasion (*P* = *1*) and those negative for nerve invasion (*P* = *0.570*) in both the high and low-ECPI groups, as well as in the group with negative and positive vascular signs (Fig. 5B). However, compared with the low-ECPI group, the high-ECPI group exhibited higher proportions of T-stage 0–1 (82.76%, *P* < *0.001*) and N-stage 0–1 (96.55%, *P* = *0.010*). Likewise, the vascular negative group showed higher proportions of T-stage 0–1 (88.00%, *P* < *0.001*) and N-stage 0–1 (94.00%, *P* = *0.020*) (Fig. 5C).

### Impact of radiological features on postoperative recurrence and survival

**Fig. 6:**
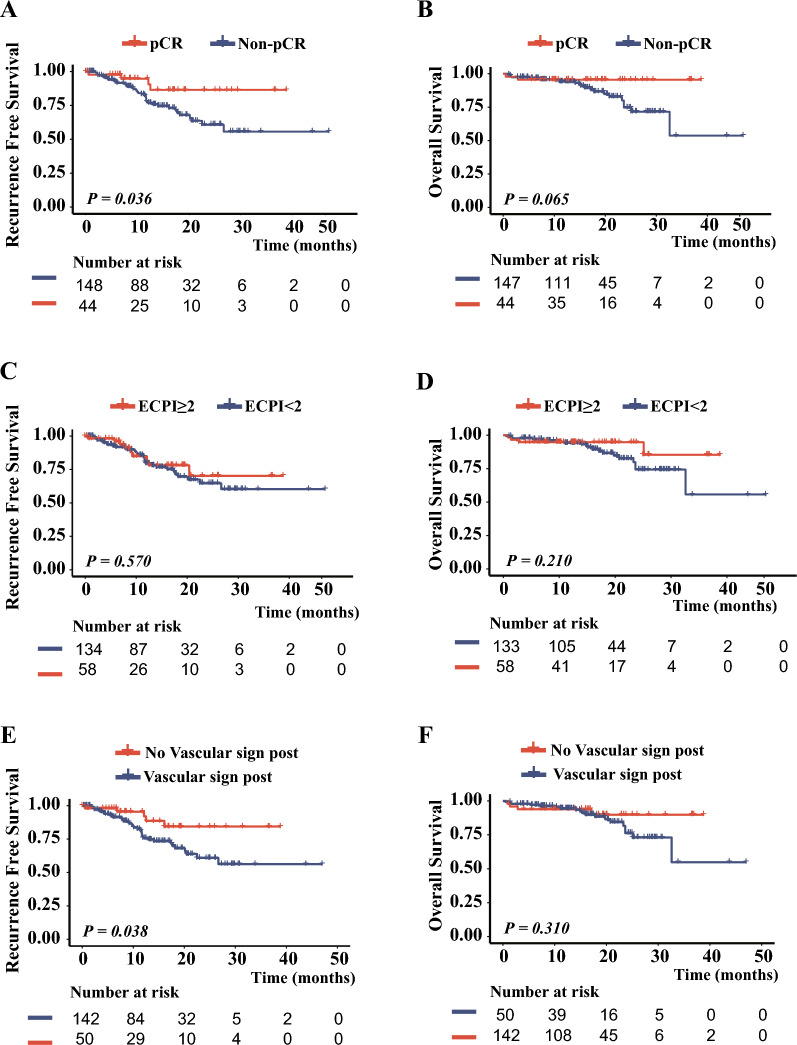
Survival analysis based on different indicators in LA-ESCC patients. **A** RFS: pCR vs. non-pCR; **B** OS: pCR vs. non-pCR; **C** RFS: score < 2 vs. score ≥ 2; **D** OS: score < 2 vs. score ≥ 2; **E** RFS: vascular sign (−) vs. vascular sign (+); **F** OS: vascular sign (−) vs. vascular sign (+)

Patients were followed up to assess time to postoperative recurrence and survival. 40 patients (20.83%) experienced disease recurrence and 23 (11.97%) patients died. Survival curves presented in Fig. 6 revealed that the pCR group exhibited better RFS (*P* = *0.036*) and OS (*P* = *0.065*) compared to the non-pCR group. Similarly, the high-ECPI group demonstrated improved RFS (*P* = *0.570*), and OS (*P* = *0.210*) compared to the low-ECPI group, and the vascular sign negative group demonstrated superior RFS (*P* = *0.038*) and OS (*P* = *0.310*) compared to the vascular sign positive group. It's worth noting that the median survivorship time was not reached due to the short follow-up period. However, patients with pCR exhibited significantly better RFS and OS compared to those with non-pCR (1-year RFS: 95% vs 77%, 2-year RFS: 86% vs 61%; 1-year OS: 95% vs 94%, 2-year OS: 95% vs 75%). Our analyses highlighted the significant association between the pCR, high-ECPI score, and the absence of vascular sign with improved survival outcomes (Table 3).

**Table 3:** Univariate and multivariate logistic regression analyses of pathological complete response in LA-ESCC

Characteristics	Univariable analyses			Multivariable analyses		
	OR	95%CI	*P.*value	OR	95%Cl	*P.*value
Sex						
Male vs female	0.59	0.05–6.65	0.67			
BMI	0.99	0.94–1.04	0.67			
Smoke						
Yes vs no	0.54	0.27–1.06	0.07			
Drink						
Yes vs no	0.42	0.21–0.84	0.01			
Cycle						
3–4 vs 1–2	0.76	0.36–1.61	0.47			
cN stages						
1 vs 0	1.75	0.63–4.88	0.29			
2 vs 0	0.55	0.16–1.93	0.35			
Unknown vs 0	0.66	0.20–2.23	0.51			
Interval time	0.81	0.45–1.47	0.49			
Tumor location						
Lower thoracic vs Cardia	3.21	0.39–26.45	0.28			
Middle thoracic vs Cardia	2.54	0.29–21.96	0.40			
ECPI-score	6.31	3.66–10.90	< 0.001	4.28	0.25–74.09	< 0.001
Vascular sign						
Yes vs no	0.02	0.01–0.06	< 0.001	0.12	0.01–2.01	< 0.001
Lymph node size	1.04	1.00–1.09	0.07			

*ECPI-score* ESCC preoperative imaging score

### Correlation between tumor regression grade and radiological features

We selected a total of 92 patients whose pathological findings included tumor regression grade (TRG). According to the Mandard TRG criteria, marked TRG 1–3 as a good pathological response was contrasted with TRG 4 or 5 as a poor pathological response [30]. 47.82% (44/92) of patients were assessed as TRG 1 (pathological complete response, pCR), 14.13% (13/92) as TRG 2, 16.30% (15/92) as TRG 3, 15.22% (14/92) as TRG 4, and 5.43% (6/92) as TRG 5. As shown in the baseline sTable3, ECPI-Score, pCR, vascular sign, postoperative TN staging, metastatic lymph node size and vascular invasion were associated with TRG grading (P < 0.001). TRG grades 1–3 predominantly occur in the high-ECPI group and vascular sign-negative groups. Specifically, in the high-ECPI group, TRG 1–3 accounted for 97.96%, while TRG 4–5 constituted only 2.33% (sFigure 1A). Similarly, within the cohort exhibiting negative vascular sign, TRG grades 1–3 comprised 95%, with TRG grades 4–5 making up 5% (sFigure 1B). Furthermore, patients with TRG 1–3 demonstrated significantly higher RFS and OS rates compared to those with TRG 4–5 (*P* = *0.046* and *P* = *0.007*) (sFigure 1C, D). These findings underscore both ECPI-Score and vascular sign provide some guidance on the degree of tumor regression.

## Discussion

The retrospective study aimed to predict pathological response and prognosis after NICT in patients with LA-ESCC by comprehensively integrating dynamic radiological features. The findings revealed significant correlations between achieving pCR and several post-treatment imaging parameters, including dynamic radiomorphology and CT attenuation values. Based on these findings, we introduced an ESCC preoperative imaging score, termed the ECPI-Score. This score offers a simple and clinically practical tool for predicting treatment outcomes and guiding subsequent therapeutic strategies.

The ECPI-Score proposed in this study demonstrates superior predictive capability (training set: AUC = 0.918, validation set: AUC = 0.862). Previous studies have focused on the value of radiomics in predicting pCR after NICT [20–25]. For example, Zhou et al. [25] used CT images from 117 patients with ESCC to extract radiomic features before NICT and esophagectomy, achieving an AUC of 0.876. Zhang et al. [23] constructed a CT-based model using pre- and post-treatment radiomics from 111 patients in the training set to predict pathological complete response (pCR). Compared with previous research, our study employs easily measurable dynamic CT imaging features, including tumor length, thickness, and CT value, both before and during preprocessing, to predict the likelihood of achieving pCR.

Cao et al. [31] demonstrated that among patients with colorectal cancer treated with neoadjuvant immunotherapy, 60.7% (n = 17) of those achieving pCR lacked vascular signs. Therefore, in addition to the ECPI-Score, we examined the association between vascular sign and treatment response in our cohort. The results suggest that patients without vascular sign after treatment are more likely to achieve pCR status, highlighting the potential utility of this imaging parameter as a predictive biomarker.

Furthermore, our study offers valuable insights into the management of LA-ESCC, particularly tumors located in anatomically challenging areas such as the neck and suprathoracic esophagus. For patients achieving pCR through NICT, active surveillance is advocated as a reliable approach for improving patient survival [32]. However, for patients with location-specific tumors that do not respond to NICT or fail to regress, additional treatment modalities such as radiotherapy may be warranted [33]. Ultimately, tailored treatment approaches based on tumor location and response to therapy are essential for optimizing patient outcomes in the management of LA-ESCC.

Despite the strengths of our study, several limitations need to be considered. These include the retrospective nature and single-center design of the study, as well as the subjective definition of the vascular sign. Future research efforts should aim to validate our findings in multi-center studies and explore the potential of artificial intelligence in overcoming limitations related to imaging resolution and observer subjectivity [34, 35].

## Conclusion

In summary, our study represents a preliminary exploration of dynamic radiological features following neoadjuvant immunochemotherapy in LA-ESCC. We developed the ECPI-Score system and identified the vascular sign, both of which were found to be significantly correlated with achieving pCR. These features hold potential as valuable tools for clinicians in identifying patients achieving pCR, especially for those considering a watch-and-wait strategy.

### Supplementary Information

Supplementary Material 1. **sFigure 1. **Comparative analysis of TRG grading and survival outcomes. (A) Distribution of TRG grading in high-ECPI and low-ECPI groups; (B) Distribution of TRG grading in vascular sign (+) and vascular sign (-) groups; (C) Survival curve analysis of Recurrence-Free Survival (RFS) for TRG 1-3 vs. TRG 4-5; (D) Survival curve analysis of Overall Survival (OS) for TRG 1-3 vs. TRG 4-5.

Supplementary Material 2.

Supplementary Material 3.

Supplementary Material 4.

## Data Availability

All data supporting the findings in this study are presented in the manuscript and the supplementary information, and additional raw data can be made available by the corresponding author upon reasonable request.
