# Supplementary figures and images for: Dynamic radiological features predict pathological response after neoadjuvant immunochemotherapy in esophageal squamous cell carcinoma
Source: J Transl Med. 2024 May 18;22:471. doi: 10.1186/s12967-024-05291-8 (PMC11102630; doi:10.1186/s12967-024-05291-8)

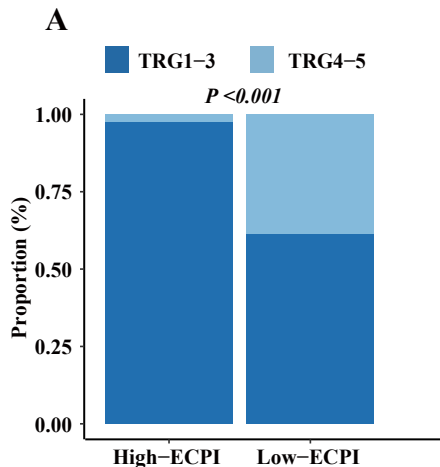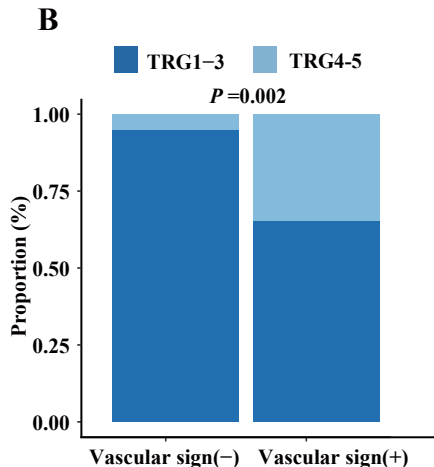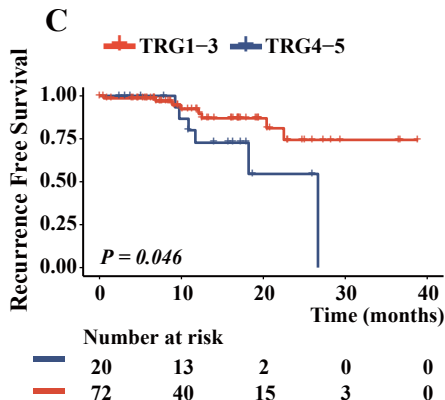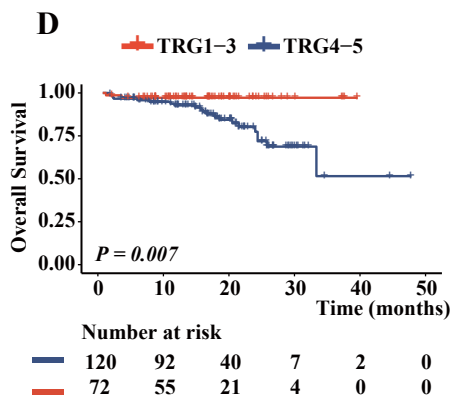

Supplement: Supplementary file 1 — Supplementary Material 1. sFigure 1. Comparative analysis of TRG grading and survival outcomes. (A) Distribution of TRG grading in high-ECPI and low-ECPI groups; (B) Distribution of TRG grading in vascular sign (+) and vascular sign (-) groups; (C) Survival curve analysis of Recurrence-Free Survival (RFS) for TRG 1-3 vs. TRG 4-5; (D) Survival curve analysis of Overall Survival (OS) for TRG 1-3 vs. TRG 4-5. [file 12967_2024_5291_MOESM1_ESM.pdf]
