# Supplementary material for: Dynamic radiological features predict pathological response after neoadjuvant immunochemotherapy in esophageal squamous cell carcinoma
Source: J Transl Med. 2024 May 18;22:471. doi: 10.1186/s12967-024-05291-8 (PMC11102630; doi:10.1186/s12967-024-05291-8)
Supplement: Supplementary file 2 — Supplementary Material 2. [file 12967_2024_5291_MOESM2_ESM.docx]

**sTable1 Interpretation of tumor-radiological features.**

| **Parameters** | **Descriptions** |
| --- | --- |
| Maximum tumor thickness | Maximum tumor thickness was measured on the axial slice with the largest tumor diameter. If the lumen is completely occluded, 1/2 of the maximum diameter of the tumor in the largest tumor section was considered. as the maximum tumor thickness. |
| Maximum tumor length | The maximum tumor length in the superoinferior direction was measured on the sagittal planar images obtained by multiplanar reformation. To accurately measure the maximum tumor length on CT, esophagogram was used as the referring standard. |
| Maximum tumor area | Maximum tumor area was manually drawn on the axial slice with the largest tumor diameter, and then the tumor area was automatically calculated by the software. |
| CT Values | In the cross-sectional area with the maximum tumor size, select the region of interest and measure the corresponding CT values in that area. |
| ΔT | The difference in tumor attenuation values during the arterial phase before treatment and surgery. |
| ΔTN | The difference of CT attenuation between the tumor and background normal esophageal wall on the arterial phase images. |
| TNR | The CT attenuation value ratio of the tumor to background normal esophageal wall on the arterial phase images. |
| Max intensity CT value | Draw a region of interest on the cross-sectional view of the tumor and select the maximum CT attenuation value within the chosen area. |
| Vascular sign | striated or punctate vessels remaining within the radiological residual tumor in CT images of arterial phase. |

**Notes：**CT, computed tomography.
