# Supplementary material for: Dynamic radiological features predict pathological response after neoadjuvant immunochemotherapy in esophageal squamous cell carcinoma
Source: J Transl Med. 2024 May 18;22:471. doi: 10.1186/s12967-024-05291-8 (PMC11102630; doi:10.1186/s12967-024-05291-8)
Supplement: Supplementary file 3 — Supplementary Material 3. [file 12967_2024_5291_MOESM3_ESM.docx]

**sTable2 Radiological features comparison of sensitivity, specificity, AUC area, and P value.**

| **Variable** | **Cut-off** | **Sensitivity** | **Specificity** | **AUC** | ***P.value*** |
| --- | --- | --- | --- | --- | --- |
| **Max intensity CT post** | **69.500** | **0.818** | **0.899** | **0.900** | **<0.001** |
| **Percentage change in tumor maximum thickness** | **0.511** | **0.591** | **0.912** | **0.810** | **<0.001** |
| **Maximum tumor thickness post** | **8.500** | **0.659** | **0.818** | **0.808** | **<0.001** |
| **Percentage change in tumor maximum length** | **0.371** | **0.750** | **0.764** | **0.765** | **<0.001** |
| Percentage change in tumor maximum area | 0.626 | 0.705 | 0.703 | 0.739 | <0.001 |
| Maximum tumor length post | 36.500 | 0.773 | 0.635 | 0.734 | <0.001 |
| Maximum tumor area post | 176.500 | 0.568 | 0.764 | 0.700 | <0.001 |
| CT values post | 43.750 | 0.545 | 0.811 | 0.689 | <0.001 |
| ΔT | 13.750 | 0.568 | 0.750 | 0.674 | <0.001 |
| ΔTN post | 19.250 | 0.432 | 0.804 | 0.653 | 0.329 |
| TNR post | 2.166 | 0.659 | 0.527 | 0.596 | 0.002 |
| Max intensity CT pre | 85.500 | 0.705 | 0.419 | 0.565 | 0.188 |
| Maximum tumor thickness pre | 18.500 | 0.432 | 0.716 | 0.564 | 0.197 |
| Maximum tumor area pre | 641.500 | 0.545 | 0.608 | 0.547 | 0.346 |
| CT values pre | 46.750 | 0.955 | 0.169 | 0.527 | 0.586 |
| Maximum tumor length pre | 54.500 | 0.523 | 0.554 | 0.485 | 0.764 |

**Notes：**ESCC, esophageal squamous cell carcinoma; pCR, pathological complete response.
