# Supplementary material for: Dynamic radiological features predict pathological response after neoadjuvant immunochemotherapy in esophageal squamous cell carcinoma
Source: J Transl Med. 2024 May 18;22:471. doi: 10.1186/s12967-024-05291-8 (PMC11102630; doi:10.1186/s12967-024-05291-8)
Supplement: Supplementary file 4 — Supplementary Material 4. [file 12967_2024_5291_MOESM4_ESM.docx]

**sTable3 Clinicopathological characteristics of 92 patients according to the Mandard TRG criteria.**

|  | **TRG=1 (n=44)** | **TRG=2 (n=13)** | **TRG=3 (n=15)** | **TRG=4 (n=14)** | **TRG=5 (n=6)** | ***P.value*** |
| --- | --- | --- | --- | --- | --- | --- |
| Age | 62.0(58.0-66.0) | 64.0 (51.0-66.0) | 60.0 (57.0-63.0) | 60.0 (57.0-63.8) | 61.0 (54.8-68.0) | 0.823 |
| Sex: |  |  |  |  |  | 0.894 |
| Female | 1 (2.27%) | 0 (0%) | 0 (0%) | 0 (0%) | 0 (0%) |  |
| Male | 43 (97.7%) | 13 (100%) | 15 (100%) | 14 (100%) | 6 (100%) |  |
| BMI | 22.6 (21.0-24.8) | 23.5 (22.0-25.3) | 24.8 (21.4-25.9) | 23.8 (20.5-24.4) | 22.1 (21.4-22.4) | 0.633 |
| Smoke: |  |  |  |  |  | 0.313 |
| No | 27 (61.4%) | 4 (30.8%) | 6 (40.0%) | 7 (50.0%) | 3 (50.0%) |  |
| Yes | 17 (38.6%) | 9 (69.2%) | 9 (60.0%) | 7 (50.0%) | 3 (50.0%) |  |
| Drink: |  |  |  |  |  | 0.807 |
| No | 29 (65.9%) | 8 (61.5%) | 8 (53.3%) | 7 (50.0%) | 4 (66.7%) |  |
| Yes | 15 (34.1%) | 5 (38.5%) | 7 (46.7%) | 7 (50.0%) | 2 (33.3%) |  |
| Circle: |  |  |  |  |  | 0.020 |
| 1-2 | 32 (72.7%) | 8 (61.5%) | 12 (80.0%) | 8 (57.1%) | 2 (33.3%) |  |
| 3-4 | 12 (27.3%) | 4 (30.8%) | 3 (20.0%) | 3 (21.4%) | 4 (66.7%) |  |
| 5-6 | 0 (0%) | 1 (7.69%) | 0 (0%) | 3 (21.4%) | 0 (0%) |  |
| cT stages: |  |  |  |  |  | 0.011 |
| 1 | 0 (0%) | 0 (0%) | 2 (13.3%) | 1 (7.14%) | 0 (0%) |  |
| 2 | 7 (15.9%) | 6 (46.2%) | 4 (26.7%) | 2 (14.3%) | 2 (33.3%) |  |
| 3 | 30 (68.2%) | 7 (53.8%) | 8 (53.3%) | 8 (57.1%) | 3 (50.0%) |  |
| 4 | 0 (0%) | 0 (0%) | 0 (0%) | 0 (0%) | 1 (16.7%) |  |
| unknown | 7 (15.9%) | 0 (0%) | 1 (6.67%) | 3 (21.4%) | 0 (0%) |  |
| cN stages: |  |  |  |  |  | 0.339 |
| 0 | 6 (13.6%) | 2 (15.4%) | 3 (20.0%) | 3 (21.4%) | 1 (16.7%) |  |
| 1 | 25 (56.8%) | 7 (53.8%) | 5 (33.3%) | 3 (21.4%) | 4 (66.7%) |  |
| 2 | 6 (13.6%) | 4 (30.8%) | 5 (33.3%) | 5 (35.7%) | 1 (16.7%) |  |
| 3 | 0 (0%) | 0 (0%) | 1 (6.67%) | 0 (0%) | 0 (0%) |  |
| unknown | 7 (15.9%) | 0 (0%) | 1 (6.67%) | 3 (21.4%) | 0 (0%) |  |
| Interval Time | 1.37 (1.15-1.58) | 1.47 (1.27-1.80) | 1.30 (1.17-1.72) | 1.48 (1.31-1.83) | 1.33 (1.12-1.50) | 0.431 |
| Tumor Location: |  |  |  |  |  | 0.040 |
| Cardia | 1 (2.27%) | 2 (15.4%) | 0 (0%) | 1 (7.14%) | 0 (0%) |  |
| Lower thoracic | 30 (68.2%) | 6 (46.2%) | 10 (66.7%) | 6 (42.9%) | 4 (66.7%) |  |
| Middle thoracic | 13 (29.5%) | 4 (30.8%) | 2 (13.3%) | 7 (50.0%) | 2 (33.3%) |  |
| Upper thoracic | 0 (0%) | 1 (7.69%) | 3 (20.0%) | 0 (0%) | 0 (0%) |  |
| ECPI-Score | 3.00 (2.00-4.00) | 1.00 (0-1.00) | 0 (0-0.50) | 0 (0-0.75) | 1.00 (0.25-1.00) | <0.001 |
| pCR: |  |  |  |  |  | <0.001 |
| 0 | 0(0.00%) | 13(100%) | 15(100%) | 14(100%) | 6(100%) |  |
| 1 | 44(100%) | 0(0.00%) | 0(0.00%) | 0(0.00%) | 0(0.00%) |  |
| Vascular sign: |  |  |  |  |  | <0.001 |
| No | 36 (81.8%) | 1 (7.69%) | 1 (6.67%) | 1 (7.14%) | 1 (16.7%) |  |
| Yes | 8 (18.2%) | 12 (92.3%) | 14 (93.3%) | 13 (92.9%) | 5 (83.3%) |  |
| Lymph node size | 10.0 (0-13.0) | 0 (0-9.00) | 9.00 (3.00-10.5) | 0 (0-10.8) | 4.50 (0-9.75) | 0.591 |
| Adjuvant therapy: |  |  |  |  |  | 0.126 |
| No | 36 (81.8%) | 8 (61.5%) | 10 (66.7%) | 7 (50.0%) | 3 (50.0%) |  |
| Yes | 8 (18.2%) | 5 (38.5%) | 5 (33.3%) | 7 (50.0%) | 3 (50.0%) |  |
| ypT stages |  |  |  |  |  | <0.001 |
| 0 | 43(97.7%) | 3(23.1%) | 0(0%) | 0(0%) | 0（0%） |  |
| 1 | 1(2.27%) | 6(46.2%) | 7(46.7%) | 2(14.3%) | 3（50%）） |  |
| 2 | 0(0%) | 2(15.4%) | 2(13.3%) | 1(7.14%) | 0（0%）） |  |
| 3 | 0(0%) | 2(15.4%) | 6(40.0%) | 11(78.6%) | 3(50.0%) |  |
| ypN stages |  |  |  |  |  | <0.001 |
| 0 | 43(97.7%) | 3(23.1%) | 4(26.7%) | 6(42.9%) | 1(16.7%) |  |
| 1 | 1(2.27%) | 8(61.5%) | 6(40.0%) | 3(21.4%) | 3(50%) |  |
| 2 | 0(0%) | 1(7.69%) | 4(26.7%) | 3(21.4%) | 2(33.3%) |  |
| 3 | 0(0%) | 1(7.69%) | 1(6.67%) | 2(14.3%) | 0(0%) |  |
| Total lymph nodes removed | 33.0 (24.0-53.2) | 35.0 (30.0-62.0) | 52.0 (38.5-60.0) | 29.5 (23.8-46.0) | 39.0 (31.0-44.0) | 0.267 |
| Number of metastatic lymph | 0 (0-0) | 1.00 (1.00-2.00) | 2.00 (0.50-3.50) | 1.00 (0-3.75) | 2.00 (1.25-2.75) | <0.001 |
| Vascular invasion: |  |  |  |  |  | <0.001 |
| No | 44 (100%) | 13 (100%) | 14 (93.3%) | 14 (100%) | 4 (66.7%) |  |
| Yes | 0 (0%) | 0 (0%) | 1 (6.67%) | 0 (0%) | 2 (33.3%) |  |
| Nerve invasion: |  |  |  |  |  | 0.268 |
| No | 44 (100%) | 13 (100%) | 14 (93.3%) | 14 (100%) | 6 (100%) |  |
| Yes | 0 (0%) | 0 (0%) | 1 (6.67%) | 0 (0%) | 0 (0%) |  |

**Notes:** pCR, pathological complete response; ECPI-Score, ESCC preoperative imaging score.
